# Spatial income polarization and all-cause mortality: Local environmental attributes as mediators

**DOI:** 10.1093/geroni/igaf122.2728

**Published:** 2025-12-31

**Authors:** Ethan Siu, Leung Cheung, David S Curtis

**Affiliations:** The University of Hong Kong, Hong Kong; University of Utah, Salt Lake City, Utah, United States

## Abstract

Although numerous studies have reported associations between segregation and mortality, the contextual effect of income segregation, in comparison to racial segregation, is less researched. Furthermore, the extent to which local environmental features mediate this relationship is not well understood. This study examines the association between spatial income polarization with all-cause mortality, and tests local environmental features as mediators. We adopt individual data from three waves of the core sample in the Midlife in the United States study, supplemented with an oversample of African Americans from Milwaukee: Wave 1 (1995-96), Wave 2 (2004-06), and Wave 3 (2016-17). The mortality data was observed until 2022 from the National Death Index and supplemental field tracing. Our sample includes 6,460 participants with 1580 deaths throughout the study period. Spatial income polarization is measured by the Index of Concentration at the Extremes (ICE) at the tract level, further adjusted by regional price parity index. The local environmental features include area-level economic disadvantage, neighborhood trust, neighborhood safety, neighborhood disorder, PM10 concentration, heat island days, and accessibility to greenspace. Survival models with a Weibull distribution are employed to examine the associations. Results showed that one-unit increase of ICEincome is associated with 32% lower risk of mortality, highlighting the health advantage of individuals living in more income-privileged relative to income-deprived tracts. Adjustments for all environmental features, including neighborhood safety and PM10 concentration, attenuated the coefficient of ICEincome by 63%. The findings underscore the importance of the local environment in explaining the relationship between tract income concentration and mortality.

